# Poster Session II - A302 RISK FACTORS FOR THE DEVELOPMENT AND PROGRESSION OF PERIANAL CROHN’S DISEASE: A SYSTEMATIC REVIEW

**DOI:** 10.1093/jcag/gwaf042.302

**Published:** 2026-02-13

**Authors:** J Zhen, S Gupta, S Xiu, A Datta, A Stojanova, P Cooper, J McCurdy

**Affiliations:** University of Ottawa, Ottawa, ON, Canada; University of Ottawa, Ottawa, ON, Canada; University of Ottawa, Ottawa, ON, Canada; McGill University, Montreal, QC, Canada; University of Ottawa, Ottawa, ON, Canada; University of Toronto, Toronto, ON, Canada; University of Ottawa, Ottawa, ON, Canada

## Abstract

**Background:**

Perianal fistulizing Crohn’s disease (pfCD) is a highly heterogenous phenotype of Crohn’s disease ranging from self-limited to severe recalcitrant disease requiring completion proctectomy. Our current understanding of risk factors for the development and progression of pfCD remain poorly defined.

**Aims:**

The aim of this study is to identify the risk factors associated with 1) the development of pfCD and 2) the progression of pfCD in patients with Crohn’s disease.

**Methods:**

We performed a systematic review in accordance with PRISMA guidelines and an a priori protocol registered in the Open Science Framework. MEDLINE, EMBASE, Web of Science, and the Cochrane Central Register of Controlled Trials were searched through to October 2025. Eligible studies included cohort, case-control, population-based studies, and randomized controlled trials reporting either variables associated with the risk of development or progression of pfCD. We defined the risk of progression as lack of response/remission to therapy or requirement for fecal diversion.

**Results:**

A total of 101 studies evaluating risk factors for pfCD development and 88 studies assessing pfCD progression were included. Across studies, demographic factors including male sex, younger age at diagnosis, longer disease duration, and disease-related factors including rectal involvement, colonic disease, and penetrating phenotype were frequently associated with the development of pfCD. In contrast, smoking, BMI, and extra-intestinal manifestations showed limited and conflicting evidence. For disease progression, prior intestinal surgery, greater fistula length, and fistula complexity demonstrated the most consistent association with poor outcomes, whereas younger age at diagnosis, longer disease duration, and penetrating phenotype were occasionally linked to an adverse prognosis.

**Conclusions:**

In this systematic review, male sex, longer disease duration, colonic disease, and a luminal penetrating phenotype were identified as potential risk factors for the development of pfCD, while prior intestinal surgery and fistula complexity were identified as potential risk factors for disease progression. Future work will quantify the magnitude of risk of each of these variables through meta-analytics.

**Funding Agencies:**

None

